# Use of Ebola Vaccines — Worldwide, 2021–2023

**DOI:** 10.15585/mmwr.mm7316a1

**Published:** 2024-04-25

**Authors:** Ruth Kallay, Reena H. Doshi, Pierre Muhoza, Mary J. Choi, Anaïs Legand, Emma Aberle-Grasse, Aminata Bagayoko, Terri B. Hyde, Pierre Formenty, Alejandro Costa

**Affiliations:** ^1^Global Immunization Division, Global Health Center, CDC; ^2^Emergency Preparedness and Response, World Health Organization Regional Office for Africa, Brazzaville, Republic of the Congo; ^3^Division of High-Consequence Pathogens and Pathology, National Center for Emerging and Zoonotic Infectious Diseases, CDC; ^4^Viral Hemorrhagic Fevers team, Health Emergencies Programme, World Health Organization, Geneva, Switzerland.

**Summary**

**What is already known about this topic?**

The International Coordinating Group on Vaccine Provision established an Ebola vaccine stockpile in 2021 to ensure equitable, rapid access to vaccines during an outbreak.

**What is added by this report?**

Since 2021, the absence of large Ebola virus disease (Ebola) outbreaks has resulted in fewer vaccine doses being used for outbreak response. Out of the 145,690 doses shipped from the stockpile through 2023, 95% (139,120) have been repurposed for preventive vaccination, and 5% (6,570) were used in outbreak response.

**What are the implications for public health practice?**

Repurposing doses for preventive vaccination could be prioritized in the absence of Ebola outbreaks to prevent transmission and maximize the cost-efficiency and benefits of the stockpile.

## Abstract

Ebola virus disease (Ebola) is a rare but severe illness in humans, with an average case fatality rate of approximately 50%. Two licensed vaccines are currently available against *Orthoebolavirus zairense*, the virus that causes Ebola: the 1-dose rVSVΔG-ZEBOV-GP (ERVEBO [Merck]) and the 2-dose regimen of Ad26.ZEBOV and MVA-BN-Filo (Zabdeno/Mvabea [Johnson & Johnson]). The Strategic Advisory Group of Experts on Immunization recommends the use of 1-dose ERVEBO during Ebola outbreaks, and in 2021, a global stockpile of ERVEBO was established to ensure equitable, timely, and targeted access to vaccine doses for future Ebola outbreaks. This report describes the use of Ebola vaccines and the role of the stockpile developed and managed by the International Coordinating Group (ICG) on Vaccine Provision during 2021–2023. A total of 145,690 doses have been shipped from the ICG stockpile since 2021. However, because outbreaks since 2021 have been limited and rapidly contained, most doses (139,120; 95%) shipped from the ICG stockpile have been repurposed for preventive vaccination of high-risk groups, compared with 6,570 (5%) used for outbreak response. Repurposing doses for preventive vaccination could be prioritized in the absence of Ebola outbreaks to prevent transmission and maximize the cost-efficiency and benefits of the stockpile.

## Introduction

*Orthoebolavirus zairense*, the virus responsible for Ebola virus disease (Ebola), has caused the largest filovirus outbreaks worldwide; the average Ebola case fatality rate is approximately 50% (*1*). Currently, two licensed vaccines are recommended for the prevention of Ebola caused by *Orthoebolavirus zairense*: the 1-dose rVSVΔG-ZEBOV-GP (ERVEBO [Merck]) and the 2-dose Ad26.ZEBOV and MVA-BN-Filo (Zabdeno/Mvabea [Johnson & Johnson]) (*2*). ERVEBO was licensed by the European Medicines Agency and the Food and Drug Administration in 2019 and is indicated for use in persons aged >12 months (*2*,*3*). It has a shelf life of 3 years. The vaccine has also been approved in Burundi, Central African Republic, Côte d’Ivoire, Democratic Republic of the Congo (DRC), Ghana, Guinea, Republic of the Congo, Rwanda, Sierra Leone, Uganda, and Zambia (Merck regulatory department, personal communication, December 6, 2023) (*2*). In 2021, the Strategic Advisory Group of Experts on Immunization recommended using ERVEBO in ring vaccination during Ebola outbreaks, because it confers protection after 1 dose (*4*). Zabdeno/Mvabea is recommended for preventive vaccination in areas at lower risk for Ebola (or areas neighboring an outbreak) because the full regimen requires 2 doses administered 56 days apart (*4*).

ERVEBO was shown to be safe and effective during clinical trials and has likely played an important role in limiting Ebola morbidity and mortality during outbreaks since it was first introduced (*2*). In a study conducted in Ebola treatment facilities in DRC, 56% of unvaccinated patients died from Ebola, compared with 25% of patients vaccinated before symptom onset (*5*). Ensuring timely availability of Ebola vaccine doses in the event of a major Ebola outbreak is crucial to limiting its spread and protecting global health security.

In 2021, a global stockpile of ERVEBO was established under the International Coordinating Group (ICG) on Vaccine Provision to ensure equitable and timely access to vaccine doses for Ebola outbreaks* (*6*). Upon the establishment of the ICG stockpile, the global agreement was to maintain the stockpile at 500,000 doses (*6*). Gavi, the Vaccine Alliance (https://www.gavi.org), supports the procurement of vaccine and operational costs to countries for vaccination (*6*). Whereas the availability of doses for outbreak response is the primary objective of the stockpile, ICG has approved requests for targeted preventive vaccination of high-risk groups, including health care workers and frontline workers in countries at risk for Ebola outbreaks. This report describes the use of Ebola vaccines and the role of the ICG vaccine stockpile during 2021–2023.

## Methods

Data on past Ebola outbreaks were obtained from the World Health Organization (WHO) Regional Office for Africa’s weekly Outbreak and Emergencies situation reports (*1*). Information on Ebola vaccine stockpile requests and deliveries during 2021–2023 was obtained from the ICG Secretariat. Data on the stockpile size were obtained from UNICEF Supply Division’s ICG Ebola vaccine stockpile report dated January 19, 2024 (*7*). This activity was reviewed by CDC, deemed not research, and was conducted consistent with applicable federal law and CDC policy.^†^

## Results

Ebola vaccine was first used during clinical trials in the 2014–2015 West African outbreak, then under a compassionate use protocol in Guinea during 2015, and again in the 2018–2020 eastern DRC outbreak. Since 2015, when Ebola vaccines were first deployed in outbreak response, recorded Ebola outbreaks have varied in frequency, size, and origin, with recent outbreaks more often linked to reintroduction through viral persistence^§^ (four of five outbreaks since 2021) than to zoonotic spillover (Table 1).

**TABLE 1:** Characteristics of reported Ebola virus disease outbreaks — World Health Organization, African Region, 2014–2023*

Start date–end date	Total no. of cases	Total no. of deaths (CFR, %)	Country or countries	Region/Province	Origin
Mar 23, 2014–Jun 10, 2016	**28,610**	**11,308 (40)**	Guinea, Liberia, and Sierra Leone	NA	Zoonotic spillover
Jul 23–Oct 20, 2014	**20**	**8 (40)**	Nigeria	Lagos	Human transmission from West Africa outbreak
Jul 26–Nov 21, 2014	**69**	**49 (71)**	DRC	Equateur	Zoonotic Spillover
Aug 23–Oct 17, 2014	**8**	**6 (75)**	Senegal	Dakar	Human transmission from West Africa outbreak
Oct 23–Dec 6, 2014	**1**	**0 (—)**	Mali	Bamako and Kayes	Human transmission from West Africa outbreak
May 11–Jul 2, 2017	**8**	**4 (50)**	DRC	Bas Uele	Zoonotic spillover
May 8–Jul 24, 2018	**54**	**33 (61)**	DRC	Equateur	Zoonotic spillover
Jun 1–Nov 18, 2020	**130**	**55 (42)**	DRC	Equateur	Zoonotic spillover and viral persistence
Aug 1, 2018–Jun 25, 2020	**3,470**	**2,287 (66)**	DRC and Uganda	North Kivu, South Kivu, and Ituri	Zoonotic spillover
Feb 7–May 3, 2021	**12**	**6 (50)**	DRC	North Kivu	Viral persistence
Feb 14–Jun 19, 2021	**23**	**12 (52)**	Guinea	N'Zérékoré	Viral persistence
Oct 8–Dec 16, 2021	**11**	**9 (82)**	DRC	North Kivu	Viral persistence
Apr 23–Jul 4, 2022	**5**	**5 (100)**	DRC	Equateur	Zoonotic spillover^†^
Aug 22–Sep 27, 2022	**1**	**1 (100)**	DRC	North Kivu	Viral persistence^§^

**Abbreviations:** CFR = case fatality rate; DRC = Democratic Republic of the Congo; NA = not applicable.

* Outbreak data obtained from the World Health Organization Regional Office for Africa weekly Outbreak and Emergencies situation reports was compared with data from CDC available online. https://www.cdc.gov/vhf/ebola/history/chronology.html (Accessed January 9, 2024).

^†^ Zoonotic spillover is the transmission of virus from an animal to a human.

^§^ Person-to-person transmission of Ebola virus from virus that persisted in immunologically privileged sites (sites that are able to tolerate the introduction of antigen without eliciting an inflammatory immune response, including the eyes, placenta, fetus, testicles, and central nervous system) or body fluids after recovery from acute infection.

The ICG Ebola vaccine stockpile reached the goal of 500,000 doses in 2022 and, as of December 2023, holds 518,890 doses. In total, 208,390 (40%) doses from the current stockpile are scheduled to expire in 2024. Doses from the ICG stockpile were first deployed in 2021 in DRC for outbreak response. During 2021–2023, a total of 145,690 ERVEBO doses were shipped through requests from the ICG stockpile. Among 11 requests to ICG during this period, 10 were approved or partially approved, and one request was declined^¶^ (Table 2). All requests to ICG for outbreak response (three of 11) were delivered within 1 week of being received. Longer times to delivery were noted for shipments intended for preventive vaccination because of the additional planning and engagement around those activities.

**TABLE 2:** Requests to the International Coordinating Group on Vaccine Provision for Ebola vaccine deliveries from global stockpile, by country and year — worldwide, 2021–2023

Country	Year	No. of doses requested	No. of doses shipped	Vaccination strategy	Target groups	Days from request to delivery	Approval status
**DRC**	2021	4,800	4,800	Outbreak response	Ring vaccination	6	Approved
	2022	1,570	1,770	Outbreak response	Ring vaccination	7	Approved
	2022	962	962*	Outbreak response	Ring vaccination	2	Approved
	2023	75,000	21,670	Preventive campaign	Frontline workers^†^	20	Approved
	2023	82,647	82,760	Preventive campaign	Frontline workers	30	Approved
**Uganda**	2022	12,000	12,060	Preventive campaign	Frontline workers	25	Approved
	2023	17,096	11,400	Preventive campaign	Frontline workers and security forces	118	Partially approved
**Guinea-Bissau**	2023	10,963	11,170	Preventive campaign	Health care and frontline workers and support staff members	48	Approved
**Switzerland**	2022	40	40	Preventive campaign	International frontline workers	0	Approved
	2023	20	20	Preventive campaign	International frontline workers	0	Approved
**Kenya**	2022	2,000	0	Preventive campaign	Security forces	NA	Not approved^§^

**Abbreviations:** DRC = Democratic Republic of the Congo; ICG = International Coordinating Group; NA = not applicable.

* Doses shifted from Equateur province to North Kivu province in DRC from previously shipped doses approved by ICG.

^†^ Frontline workers are generally considered to be personnel directly involved in essential, public-facing roles related to health services or outbreak response; countries might define this group differently.

^§^ The request to ICG that was not approved lacked justification that the security forces to be vaccinated were involved in Ebola outbreak response and were at risk. ICG invited the country to resubmit the application prioritizing staff members involved in Ebola response activities.

The number of doses shipped from the stockpile has increased annually, from 4,800 doses in 2021, to 13,870 doses in 2022, and 127,020 doses in 2023. During this period, 42,620 doses expired. Most doses shipped (139,120; 95%) were repurposed for preventive vaccination. Five percent (6,570) of doses were shipped for outbreak response use. DRC has received the largest number of vaccine doses (111,000; 76%), followed by Uganda (23,460; 16%) and Guinea-Bissau (11,170; 8%).

## Discussion

The ICG stockpile provides equitable access to vaccines that can be shipped quickly in the event of an Ebola outbreak. The relatively small number of doses used for outbreak response (6,570; 5% of doses shipped) reflects the smaller size and rapid containment of Ebola outbreaks since 2021. North Kivu, DRC, has received and administered more doses than any other geographic area worldwide since 2018, which might have contributed to the rapid containment of subsequent outbreaks in that area (*1*).

After approvals of vaccine for preventive use by ICG in 2022, WHO, in early 2023, circulated an internal memo on behalf of ICG informing at-risk countries of the availability of vaccines for preventive vaccination of health care workers and frontline workers. Preventive vaccination campaigns have targeted health care workers and frontline workers in at-risk countries, given their increased risk for exposure because of their frequent contact with patients (*8*). The addition of preventive Ebola vaccination of these workers could reduce total cases, hospitalizations, and deaths in Ebola outbreaks by an estimated 14%–38% compared with nonpharmaceutical interventions and ring vaccination alone (*8*).

The variability of Ebola outbreak size and time to containment makes predicting future vaccine needs challenging. Repurposing doses for preventive vaccination of targeted groups can protect high-risk persons as well as make use of doses with a shorter shelf life. More than 200,000 short–shelf-life doses in the ICG stockpile due to expire in 2024 could be redirected for preventive vaccination. In addition to focusing on reactive (outbreak response) vaccination, early planning for preventive vaccination with short–shelf-life doses could be incorporated into future stockpile management strategies. Additional studies accounting for the variability in outbreak size could guide planning to maximize the cost-efficiency of stockpile management.

The frequency of recent outbreaks, especially those linked to viral persistence, highlights the need for innovative strategies to protect Ebola survivors and prevent reintroductions. One such strategy is to offer postoutbreak immunization to close contacts of survivors, including new sex partners and other groups at risk for transmission because of viral persistence (*9*). Additional avenues to expand preventive vaccination among high-risk populations could be explored in countries at risk for outbreaks. Demand-generation activities** incorporating findings from community engagement and vaccine acceptance studies in targeted risk groups could accompany vaccination campaigns and help develop targeted engagement plans. Investments and advocacy for preventive vaccination against Ebola are crucial for health system preparedness and resiliency. Currently, Gavi, WHO, and UNICEF are coordinating with other partners to develop a learning agenda^††^ to help guide research prioritization and funding decisions for Ebola vaccine use.

### Limitations

The findings in this report are subject to at least two limitations. First, whereas the Ebola vaccine has reduced morbidity and mortality during outbreaks, the impact of Ebola vaccines on preventing outbreaks is difficult to ascertain because of the infrequent occurrence of the disease. Second, important data are lacking regarding the duration of protection, vaccine effectiveness in outbreak situations, and the need for booster doses. These data will be needed to guide decision-making regarding vaccination strategies and should be a focus for future research.

### Implications for Public Health Practice

The availability of licensed Ebola vaccines is an important advancement in Ebola prevention and global health security. In the absence of large-scale outbreaks, the demand for vaccines lags behind the current supply of doses, and preventive vaccination could be considered for high-risk groups. Investments, advocacy, and additional research to inform preventive vaccination are crucial for health system preparedness and resiliency. Focus on working with countries at risk for Ebola outbreaks to identify high-risk groups and generate demand for preventive vaccination is important for optimizing the use of the stockpile. Ensuring the availability of sufficient Ebola vaccine doses for emergency outbreak response remains the priority of ICG.

## Footnotes

* The ICG Ebola vaccine stockpile is managed by the ICG on Vaccine Provision comprising Médecins sans Frontières, the International Federation of Red Cross and Red Crescent Societies, UNICEF, and the World Health Organization. These organizations support maintenance and decisions regarding vaccine allocations from the ICG on Vaccine Provision’s stockpile of Ebola vaccine. https://www.who.int/groups/icg/about

† 45 C.F.R. part 46, 21 C.F.R. part 56; 42 U.S.C. Sect. 241(d); 5 U.S.C. Sect. 552a; 44 U.S.C. Sect. 3501 et seq.

§ Person-to-person transmission of Ebola virus that persisted in immunologically privileged sites (sites that are able to tolerate the introduction of antigen without eliciting an inflammatory immune response, including the eyes, placenta, fetus, testicles, and central nervous system) or body fluids after recovery from acute infection in humans, in contrast to outbreaks originating from zoonotic spillover, which is the transmission of virus from an animal to a human.

¶ The request to ICG that was not approved lacked justification that the security forces to be vaccinated were involved in Ebola outbreak response and were at risk. ICG invited the country to resubmit the application prioritizing staff members involved in Ebola response activities.

** Activities that aim to increase public awareness of and coverage with the vaccine and might include public education campaigns, health care worker education and engagement, community outreach, targeted messaging to high-risk groups, and increased access to the vaccine.

†† A set of prioritized vaccine implementation research questions and activities to guide evidence-building and decision-making around the Ebola vaccine.
